# The effect of glatiramer acetate, IFNβ-1a, fingolimod, and dimethyl fumarate on the expression of T-bet, IFN-γ, and MEG3 in PBMC of RRMS patients

**DOI:** 10.1186/s13104-023-06556-z

**Published:** 2023-10-16

**Authors:** Rozhin Dabbaghi, Reza Safaralizadeh, Shima Rahmani, Nesa Barpour, Mohammadali Hosseinpourfeizi, Ali Rajabi, Behzad Baradaran

**Affiliations:** 1 Department of Biology, Faculty of Natural Sciences, University of Tabriz, Tabriz, Iran; 2 Immunology Research Center, Tabriz University of Medical Sciences, Tabriz, Iran; 3 Department of Animal Biology, Faculty of Natural Sciences, University of Tabriz, Tabriz, Iran

**Keywords:** Multiple sclerosis, Autoimmunity, Th1 cell, B cell, LncRNA, DMTs

## Abstract

**Objective:**

Multiple sclerosis (MS) is a progressing neurodegenerative disease marked by chronic central nervous system inflammation and degeneration.This study investigates gene expression profiles of T-box transcription factor TBX21 (T-bet), interferon-gamma (IFN-γ), and long non-coding RNA MEG3 in peripheral blood mononuclear cells (PBMCs) from treatment-naïve Relapsing-Remitting Multiple Sclerosis patients (RRMS), healthy controls, and RRMS patients on different Disease Modifying Therapies (DMTs). The aim is to understand the role of T-bet, IFN-γ, and MEG3 in MS pathogenesis and their potential as diagnostic and therapeutic targets.

**Results:**

Elevated T-bet expression is observed in treatment-naïve RRMS patients compared to healthy individuals. RRMS patients treated with Interferon beta-1alpha (IFNβ-1a) and fingolimod exhibit downregulated T-bet and MEG3 expression levels, respectively, with more pronounced effects in females. Healthy individuals show a moderate positive correlation between T-bet and MEG3 and between IFN-γ and T-bet. In RRMS patients treated with Glatiramer Acetate (GA), a strong positive correlation is observed between MEG3 and IFN-γ. Remarkably, RRMS patients treated with Dimethyl Fumarate (DMF) exhibit a significant positive correlation between T-bet and MEG3. These findings underscore the diagnostic potential of T-bet in RRMS, warranting further exploration of MEG3, T-bet, and IFN-γ interplay in RRMS patients.

## Introduction

MS is an autoimmune disease characterized by chronic central nervous system inflammation [1]. The pathogenesis of MS is not fully understood, but evidence suggests that inflammation mediated by T-helper1 (Th1) and T-helper17 (Th17) cells plays a central role. Understanding T-cell biology and identifying diagnostic biomarkers related to MS can improve patient management [2–6].

Th1 cells are characterized by the production of lineage cytokine IFN-γ and T-bet [7]. Dysregulation of Th1 cells has been associated with various autoimmune disorders, including MS. IFN-γ is a significant cytokine detected in MS lesions and is produced by various immune cells. Inflammation and IFN-γ-producing Th1 response are associated with MS [8–10]. T-bet is a critical transcription factor of Th1 cells that can pave the way for Th1 differentiation and IFN-γ expression. Besides, IFN-γ facilitates the expression of T-bet and forms positive feedback [4, 11–15]. Epigenetic modifications, including long non-coding RNAs (lncRNAs) such as MEG3, have been implicated in MS development. MEG3 aberrant expression has been linked to inflammation in different tissues. [16–21]. Studies have shown that manipulating exosomes containing MiR21-5p/MEG3 affects inflammation and cell death. Inhibition of miR-21 in naïve T cells alters Th17 differentiation and promotes Th1/2-like subsets. [22, 23]. MS treatment involves various components, primarily disease-modifying therapies (DMTs), which playing a primary role in effectively reducing intensity, progression, relapses, disability, and maintain life quality [24].This study investigates the expression levels and diagnostic importance of MEG3, T-bet, and IFN-γ in untreated and treated individuals with 4 different DMTs including Glatiramer acetate (GA), Fingolimod, Dimethyl fumarate (DMF) and, Interferon beta-1alpha (IFNβ-1a). We examine these markers in healthy individuals, treatment-naïve RRMS patients, and those on various medications, exploring their diagnostic value and potential correlations.

## Materials and methods

### Study subject

The research involved 52 patients and 20 healthy individuals selected at the Tabriz Neurology Clinic in Iran. Patients, aged 20 to 60, met McDonald’s criteria (2017) [25], and had received at least two months of treatment without prior RRMS therapy. They were divided into five groups based on their treatments (GA-treated patients, fingolimod-treated patients, DMF-treated patients, IFNβ-1a- treated patients, Treatment-naïve patients). All patients were in remission during sampling. Exclusion criteria covered primary or secondary progressive MS, recent corticosteroid use, other inflammatory/autoimmune diseases, and CNS degenerative conditions. Informed written consent was obtained from all participants. The case and control groups were of the same ethnicity, and samples were collected simultaneously to minimize errors. The study’s ethics code is IR.TBZMED.REC.1399.074. For age, gender, clinicopathological information, and treatment specifics, refer to Tables 1 and 2.

**Table 1:** demographic and clinical features of study subjects

Demographic features	Patients (n = 52)	Controls (n = 20)
Gender Male n (%) female n (%)	13 (25%) 39 (75%)	6 (30%) 14 (70%)
Age (year) at time of analysis, median (range) at onset, median (range) Disease duration, median (range)	29 (17–67) 24.5 (11–63) 6 (0.1–23)	26 (18–49)
EDSS score, median (range)	3 (1–8)	
1–4 (%)	32 (62)	
4–7 (%)	17 (31)	
7$\le$ (%)	3 (7)	

**Table 2:** Treatment and gender classification

Treatment	Patients n (%)	Male n (%)	Female n (%)
IFNβ-1a	10 (19.2)	1 (10)	9 (90)
Glatiramer acetate	11 (21.2)	4 (26.4)	7 (63.6)
Fingolimod	15 (28.8)	4 (26.7)	11 (73.3)
Dimethyl fumarate	11 (21.2)	3 (27.3)	8 (72.7)
Treatment-naïve	5 (9.6)	1 (20)	4 (80)

### PBMC isolation

Peripheral blood samples (10 ml) were collected from each participant in heparinized vials. Then, density gradient centrifugation was used to separate mononuclear cells with lymphodex Ficoll (Inno-Train, Germany).

### RNA extraction and cDNA synthesis

TRIzol (GeneAll Biotechnology CO., LTD, Korea) used to extract total RNA according to the manufacturer’s instructions. After obtaining the quantitative and qualitative analysis of RNA with the Spectrophotometer (DeNovix Inc., USA), complementary DNA (cDNA) was synthesized using 2X RT-PCR Pre-Mix Taq cDNA synthesis kit (BioFact,Korea).

### Quantitative real-time PCR

We quantified the expression of T-bet, MEG3, and IFN-γ by real-time PCR (Step One Plus, Thermo Fisher Scientific, USA) using 2x Master Mix SYBR Green (Amplicon, Denmark) and specific primers for each gene. The reaction volume was 10 µl, containing 5 µl of SYBR Green Master Mix (2×), 0.5 µl of each primer pair, 3.5 µl of ddH2O, and 1 µl of cDNA (100 ng/µl). The PCR program consisted of an initial denaturation step at 94 °C for 13 min, followed by 45 cycles of denaturation at 94 °C for 10 s, annealing at 60 °C for T-bet and GAPDH, 58 °C for IFN-γ, or 57 °C for MEG3 for 30 s, and elongation at 72 °C for 20 s. The melting curves of all reactions were checked to verify the specificity of amplification. The expression levels of the target genes were normalized to GAPDH as the internal control. The primer sequences used are shown in (Table 3).

**Table 3:** The sequences of used primer

Gene		Sequence (5́ − 3́́́)
T-bet	Forward:	TCTCCTCTCCTACCCAACCAG
	Reverse:	CATGCTGACTGCTCGAAACTCA
IFN-γ	Forward:	CTCTGCATCATTTTGGGTTCT
	Reverse:	ATCCGCTACATCTGAATGACCT
MEG3	Forward:	TGGCATAGAGGAGGTGAT
	Reverse:	GGAGTGCTGTTGGAGAATA
GAPDH	Forward:	CAAGATCATCAGCAATGCCTCC
	Reverse:	GCCATCACGCCACAGTTTCC

### Statistical analysis

After conducting real-time PCR and obtaining Ct values for samples, gene expression levels in RRMS patients compared to healthy controls were determined by calculating 2^(-∆Ct) in Excel. Statistical analysis was performed using GraphPad Prism 9.0.0. Variables were analyzed using the Shapiro-Wilk normality test. Data were presented as mean ± standard errors of the mean (SEM) or median, IQR, and range. Group comparisons utilized the Kruskal-Wallis nonparametric test. Correlation analysis employed Spearman and Pearson methods based on normality test results. Diagnostic significance was assessed using ROC curves for gene expressions in treatment-naïve patients and healthy controls. A p-value < 0.05 (95% CI) indicated statistical significance.

## Results

### Differential gene expression in healthy controls, treated patients, and untreated RRMS patients

#### GA-treated patients

**Fig. 1:**
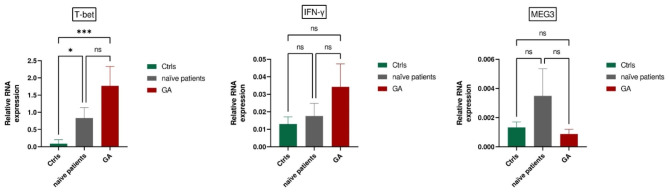
Comparative Gene Expression Profiles in RRMS Patients: T-bet Expression Significantly Elevated in Untreated RRMS Patients (P = 0.0005) and Glatiramer acetate (GA)-Treated RRMS Patients (P = 0.03) Compared to Control Group (Ctrls). No Significant Changes Observed in IFN-γ and MEG3 Expression Levels Among Groups. All Values Represent Mean ± SEM. Comparison of T-bet, IFN-γ, and MEG3 Expression Levels in Healthy Controls, Treatment-Naïve RRMS, and GA-Treated Patients; *P < 0.05, ***P < 0.001, ns: Nonsignificant

T-bet expression is significantly higher in treatment-naïve RRMS patients compared to controls (P = 0.0005). Furthermore, T-bet is upregulated in GA-treated RRMS patients (P = 0.03). However, no significant changes were observed in the expression levels of IFN-γ and MEG3 among the groups. (Fig. 1).

### Fingolimod-treated patients

**Fig. 2:**
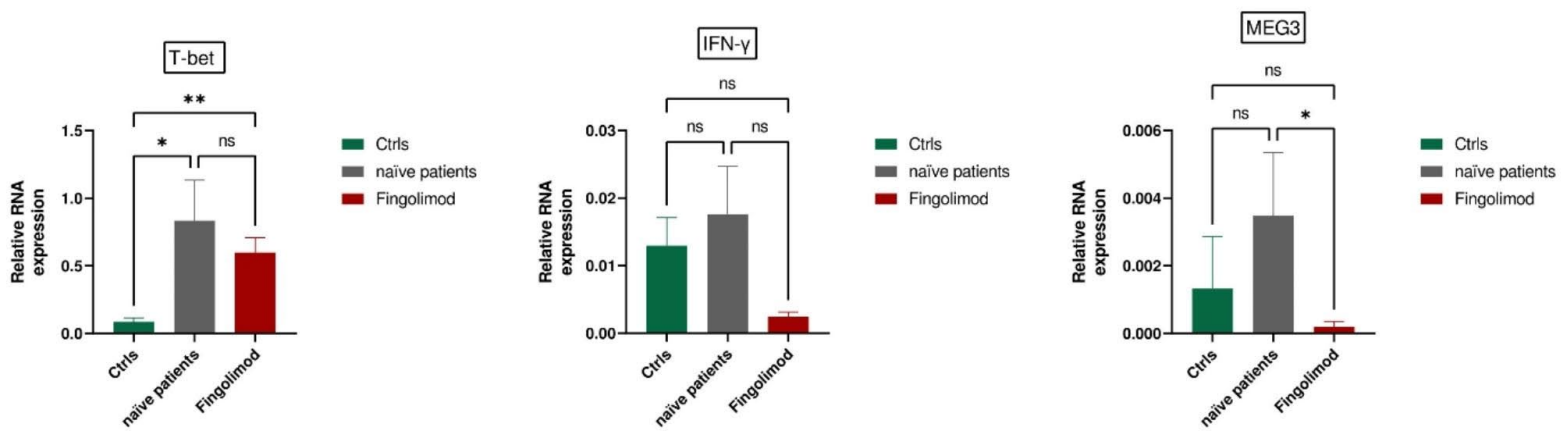
Comparative Gene Expression Profiles in RRMS Patients: Comparative analysis of T-bet, MEG3, and IFN-γ gene expressions reveals increased T-bet levels in both treatment-naïve and Fingolimod-treated RRMS patients compared to healthy controls (Ctrls) (p = 0.01, p = 0.001). Notably, MEG3 expression decreases significantly in Fingolimod-treated RRMS patients compared to the treatment-naïve group (p = 0.04), No significant changes were observed in IFN-γ expression among the groups. All Values Represent Mean ± SEM; *P < 0.05, **P < 0.01, ns: nonsignificant

T-bet expression increased in RRMS patients (both treatment-naïve and Fingolimod-treated) compared to healthy controls (p = 0.01 and p = 0.001, respectively). In contrast, MEG3 expression decreased in Fingolimod-treated patients compared to treatment-naïve RRMS patients (p = 0.04). There was no significant change observed in IFN-γ expression among the groups (Fig. 2).

### DMF-Treated patients

**Fig. 3:**
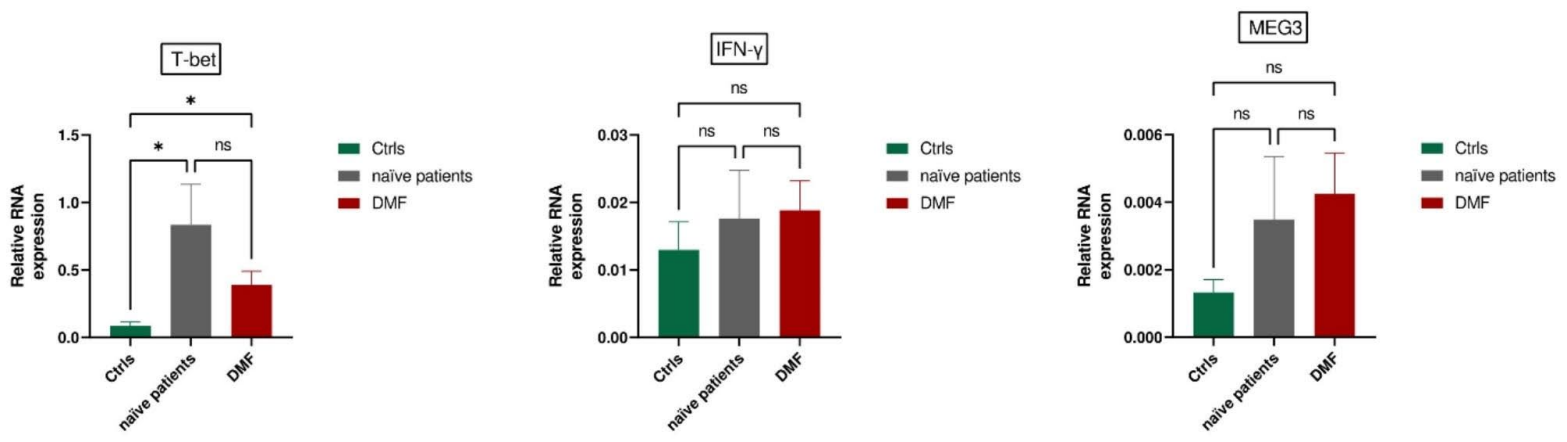
Comparative Gene Expression Profiles in RRMS Patients: The data reveal significant upregulation of T-bet expression in both treatment-naïve RRMS patients (P = 0.01) and Dimethyl fumarate (DMF)-treated patients (P = 0.02) when compared to the healthy control group (Ctrls). Conversely, no statistically significant changes were observed in the expression levels of IFN-γ and MEG3 among these groups.All Values Represent Mean ± SEM; *P < 0.05, ns: nonsignificant

The expression of T-bet is significantly upregulated in both treatment-naïve patients and DMF-treated patients when compared to healthy controls (P = 0.01 and P = 0.02, respectively). However, no statistically significant changes were observed in the expression levels of IFN-γ and MEG3 among the three groups (Fig. 3).

### IFNβ-1a -Treated patients

**Fig. 4:**
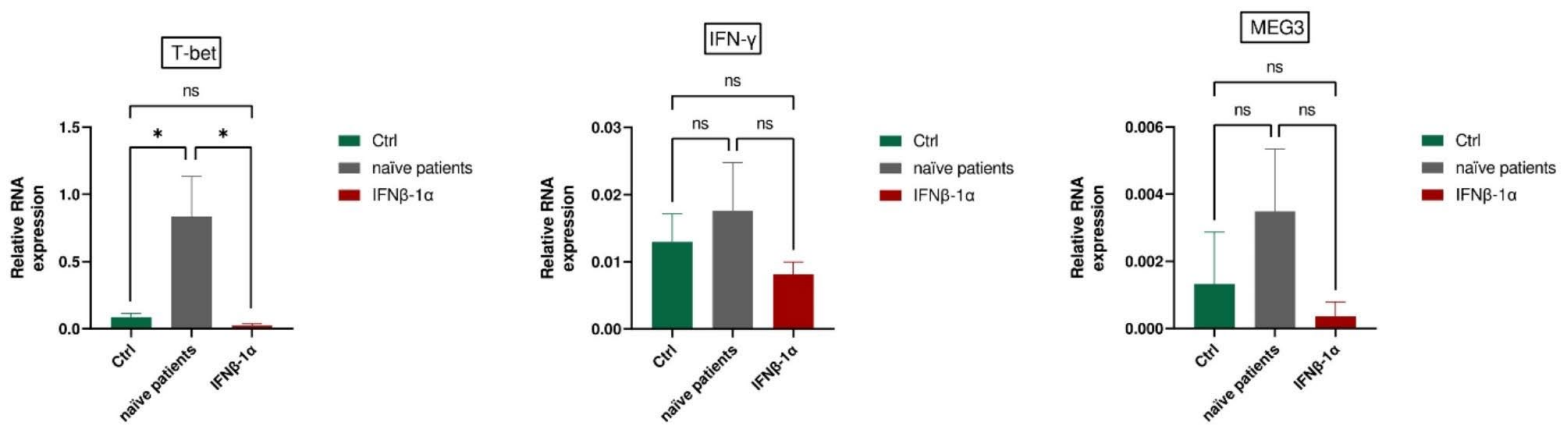
Comparative Gene Expression Profiles in RRMS Patients: T-bet Expression Significantly Elevated in Treatment-Naïve RRMS Patients Compared to Controls (Ctrls) and Interferon beta-1alpha (IFNβ-1a)-Treated Patients (P = 0.035 and P = 0.037, respectively). However, IFN-γ and MEG3 Expression Levels Show No Significant Differences Among These Groups. All Values Represent Mean ± SEM; *P < 0.05, ns: nonsignificant

T-bet expression was notably higher in treatment-naïve RRMS individuals when compared to both controls and patients treated with IFNβ-1a (P = 0.035 and P = 0.037, respectively). However, there were no significant differences observed in the expression levels of IFN-γ and MEG3 among these groups. (Fig. 4).

### Evaluation of T-bet, IFN-γ, and MEG3 expression in females with RRMS: a comparison between treated and non-treated patients

**Fig. 5:**
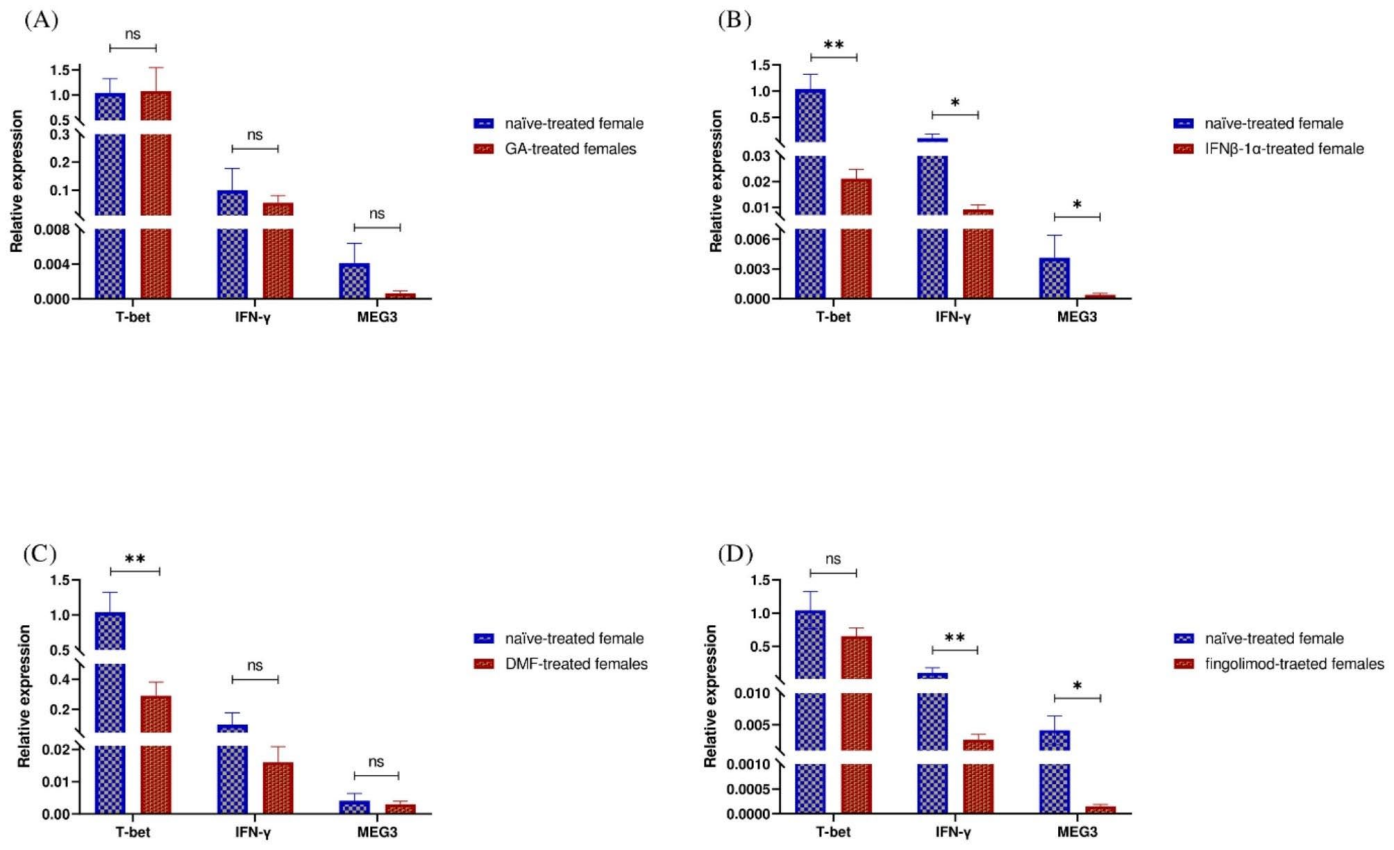
Comparative Gene Expression Profiles in female RRMS Patients: GA treatment showed no significant impact on expression levels (Fig. 5A). In contrast, IFNβ-1a treatment led to a significant reduction in T-bet, IFN-γ, and MEG3 expression (P-value = 0.004, P-value = 0.024, and P-value = 0.048, respectively) (Fig. 5B). DMF treatment resulted in a notable downregulation of T-bet expression (P-value = 0.008) (Fig. 5C), while fingolimod treatment significantly reduced IFN-γ and MEG3 expression in RRMS female patients (P-value = 0.002 and P-value = 0.011, respectively) (Fig. 5D). All Values Represent Mean ± SEM; *P < 0.05, **P < 0.01, ***P < 0.001, ns: nonsignificant

The impact of GA treatment on T-bet, IFN-γ, and MEG3 expression in female patients was found to be statistically non-significant (Fig. 5A). In contrast, IFNβ-1a treatment led to a significant reduction in the expression levels of T-bet, IFN-γ, and MEG3 (P-value = 0.004, P-value = 0.024, and P-value = 0.048, respectively) (Fig. 5B). Additionally, DMF treatment resulted in a significant downregulation of T-bet expression (P-value = 0.008) (Fig. 5C). while fingolimod treatment led to a significant downregulation of IFN-γ and MEG3 expression in female RRMS patients (P-value = 0.002 and P-value = 0.011, respectively) (Fig. 5D).

### Correlation between T-bet, IFN, and MEG3

We identified robust positive correlations among T-bet − MEG3 (r = 0.785, P < 0.0001), T-bet − IFN-γ (r = 0.68, P = 0.001), and MEG3 − IFN-γ (r = 0.753, P = 0.0003) in the cohort of healthy controls. Within the group treated with GA, a notable positive correlation emerged between MEG3 − T-bet (r = 0.691, P = 0.047). DMF-treated patients exhibited a strikingly strong positive correlation between T-bet − MEG3 (r = 0.932, P < 0.0001). Among those receiving IFNβ-1a, a positive correlation was evident between MEG3 − IFN-γ (r = 0.745, P = 0.017). Fingolimod-treated individuals displayed positive correlations across multiple pairs: T-bet − MEG3 (r = 0.715, P = 0.004), T-bet − IFN-γ (r = 0.664, P = 0.009), and MEG3 − IFN-γ (r = 0.772, P = 0.001). However, no significant correlations were observed among the studied genes in treatment-naïve patients (Table 4).

**Table 4:** Correlation between the genes expressed in different groups

Participants	T-bet, MEG3		IFN-γ, T-bet		MEG3, IFN-γ	
	r	P	r	P	r	P
Controls	0.892*	< 0.0001	0.68*	0.001	0.753*	0.0003
IFNβ-1a-treated	0.632	0.06	0.268	0.45	0.745*	0.017
GA-treated	0.691*	0.023	0.264	0.435	0.591	0.061
Fingolimod-treated	0.715*	0.004	0.664*	0.009	0.772*	0.001
DMF-treated	0.932*	< 0.0001	0.564	0.076	0.555	0.081
Treatment-naïve	0.059	0.925	-0.104	0.867	0.8266	0.084

* Significant correlation

### The diagnostic value of T-bet, IFN-γ, and MEG3 in RRMS

**Fig. 6:**
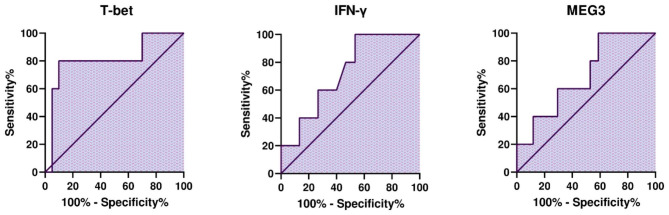
The diagnostic values of T-bet, MEG3, and IFN-γ in RRMS patients: T-bet as a Promising Diagnostic Marker for Treatment-Naïve RRMS Patients. T-bet exhibits strong diagnostic potential with an AUC of 0.81 (P-value = 0.03), surpassing IFN-γ and MEG3. The optimal cutoff point (> 0.6135, 95% CI = 0.5636 to 1.000) offers a sensitivity of 80.00% and specificity of 90.00%

T-bet exhibits significant diagnostic potential (AUC = 0.81, P-value = 0.03) in the identification of treatment-naïve RRMS patients. The optimal cutoff point is > 0.6135 (95% CI = 0.5636 to 1.000). In contrast, IFN-γ and MEG3 do not possess diagnostic value (Table 5; Fig. 6).

**Table 5:** ROC curve statistical evaluation for biomarker efficacy in MS patients

ROC curve details	T-bet	IFN-γ	MEG3
AUC	0.81	0.73	0.69
Cut-off point	> 0.6135	> 0.001780	> 0.000185
95% CI	0.5636 to 1.000	0.4915 to 0.9619	0.4442 to 0.9440
Std. error	0.1257	0.1200	0.1275
Sensitivity%	80.00	100.0	100.0
Specificity%	90.00	46.67	41.18
P-value	0.0352	0.1378	0.1961

## Discussion

The intricate web of gene expression patterns unveiled by this study within the context of RRMS offers a compelling insight into the multifaceted nature of the disease and its intricate responsiveness to diverse therapeutic interventions. A critical analysis of the results reveals both consistencies and discrepancies when compared to existing research, shedding light on the complex interplay between gene regulation and the pathophysiological processes underlying RRMS.

The identification of T-bet as a potential diagnostic marker for treatment-naïve RRMS patients aligns with previous studies that emphasize the role of T-bet in autoimmune responses [26, 27]. This finding is particularly noteworthy as it provides a promising avenue for the early identification of patients who may benefit from specific therapeutic strategies.

The observed positive correlations between T-bet and other genes (MEG3, IFN-γ) in healthy controls prompt an exploration into the regulatory interplay between these genes. This alignment is consistent with the known role of T-bet in orchestrating immune responses [28, 29]. However, the notable absence of similar correlations in treatment-naïve RRMS patients raises intriguing questions about the distinct immune dynamics at play in this subgroup.

A meticulous comparison of the gene correlations in DMT-treated patients underscores the intricate nature of their responses. While the study establishes correlations between T-bet and MEG3, and IFN-γ in these groups, the lack of such correlations in treatment-naïve patients poses a stark contrast. This discrepancy might be attributed to variations in immunological milieu observed between patients commencing Disease-Modifying Therapy (DMT) and those who remain treatment-naïve, possibly implicating distinct mechanisms of immune modulation [30].

Further analysis of gender-specific treatment responses unveils nuances in the effects of GA and IFNβ-1a. The lack of significant effects of GA on gene expression in female patients echoes its established immune-modulating mechanisms, which are less gender-dependent. Conversely, the substantial reduction of T-bet, IFN-γ, and MEG3 expression by IFNβ-1a aligns with its known anti-inflammatory properties and its capacity to modulate immune responses by reducing the expression levels of proinflammatory cytokines such as IFN-γ Comparisons with existing literature reveal a mix of consistencies and disparities suggesting that the effects of these medications on gene expression and gender-specific responses are complex and may vary in different studies or patient populations [24, 31, 32]. The elevated expression of T-bet in RRMS patients, particularly those undergoing Fingolimod treatment, is in line with prior studies highlighting T-bet’s role in inflammation. However, the unexpected downregulation of MEG3 in Fingolimod-treated patients contradicts some studies that underscore MEG3’s potential as an immunomodulatory factor, High MEG3 expression has been linked to immune hallmarks like Inflammatory Response and IFN-γ Response [20, 33]. These divergent results may stem from variations in study design, patient characteristics, or treatment durations.

The limitations of the study, including the inability to isolate Th1 cells, the relatively small sample size for male-focused analysis, and the modest representation of treatment-naïve RRMS patients, are transparently acknowledged. These limitations underscore the importance of considering the study’s findings within the context of these constraints and highlight avenues for future research to address these gaps. this study offers a comprehensive exploration of gene expression dynamics in RRMS patients and their responses to diverse therapeutic approaches. Through a rigorous comparison of the study’s outcomes with existing research, we reveal both alignments and divergences, underscoring the complex nature of RRMS pathophysiology. This study stands as a significant contribution to the field, providing valuable insights that contribute to our understanding of disease progression and treatment outcomes. Further investigations into the mechanistic underpinnings of gene interactions and their implications will be pivotal in refining therapeutic strategies and deepening our comprehension of RRMS complexities.

## Conclusion

The study sheds light on the interplay of gene expression patterns in RRMS, enhancing our understanding of immune dysregulation. It provides insights into T-bet, MEG3, and IFN-γ dynamics across diverse treatments, with T-bet being a potential diagnostic biomarker for RRMS. This research lays a foundation for refined therapeutic strategies and deepens our comprehension of RRMS intricacies for improved patient outcomes.

## Data Availability

The datasets analyzed during the current study are available from the corresponding author on reasonable request.

## Abbreviations

RRMS: Relapsing-Remittng Multiple sclerosis
Th1: T Helper 1
Th17: T Helper 17
Th2: T Helper 2
LncRNA: Long non coding Ribonucleic acid
MEG3: maternally expressed gene 3
PBMC: peripheral blood mononuclear cell
T-bet: T-box expressed in T cells
IFN-γ: Interferon gamma
GA: Glatiramer Acetate
IFNβ-1a: Interferon beta-1a
DMF: Dimethyl Fumarate
DMT: Disease-Modifying Therapy
SEM: Standard Error of the Mean
IQR: interquartile range
ROC curve: The Receiver Operating Characteristics curve
AUC: The Area Under the Curve
GM-CSF: Granulocyte-Macrophage Colony-Stimulating Factor
EAE: Experimental Autoimmune Encephalomyelitis
